# Department of Public Health and Vital Statistics, State of Texas

**Published:** 1908-05

**Authors:** 


### Department of Public Health.

---

DEPARTMENT OF PUBLIC HEALTH AND VITAL STATISTICS,
STATE OF TEXAS.

AUSTIN, TEXAS, April 25, 1908.

*To the Physicians of Texas:*

I am Chairman of Section on State Medicine at the coming session of the State Medical Association, which meets in Corpus Christi, May 12th to 14th. We have prepared an interesting program and each subject has special reference to some of our present or contemplated work. Again, the author of each paper has some official capacity and is well acquainted with the subject at hand.

The next most appropriate thing is the right kind of an audience, and we want all health officers possible to attend the S. M. A. and particularly this section. The time assigned us is Wednesday, 9-12 a. m. Don't forget the day, Wednesday.

Another thing I would like to bring to mind is the fact that we have an opportunity to get together in the same hotel at very reasonable rates in case you wish to take advantage of it. The Epworth League grounds, on the most delightful beach in the world, is close at hand and the hotel will be open on May 1st. The management has offered us lodging and breakfast for $1.00 per day and the privileges of the bath house thrown in.

There are only sixty-six rooms, and while most of them have double beds, yet this will not entertain all our health officers, for we have nearly 400 enrolled. So any of you that wish to reserve a bed please let me know as early as possible, for it will have to be "first come, first served." In case you are expecting to bring your wife, don't fail to mention it. By the way, fishing is good, bathing is better and boating is the best yet. In case there is no yellow fever in sight at that time it is possible one or two of our biggest and best quarantine boats will be in hailing distance, but don't mention it for fear some of the balance will wish they were health officers.

Again, "dollar dinners" are popular just now, so what's the matter with our having a dinner Thursday, the last evening of the meet? Instead of toasts, we might "talk shop"—relate some of our experiences, while giving our digestive apparatus an opportunity to assimilate each course.

Hoping that you will be able to join us and will so state in your next communication, I remain

Yours very truly,
W. M. BRUMBY,
State Health Officer.

---

AUSTIN, TEXAS, April 25, 1908.

*To the County Health Officer:*

DEAR DOCTOR: My request to the health officers to meet at Corpus Christi met with so much encouragement, and there will be apparently such a large attendance, that I have decided to have the Annual Conference of Boards of Health and Health Officers on Friday, May 15th, immediately following the annual session of the State Medical Association, the meeting to convene at 9 a. m. at the Auditorium in the Epworth League grounds.

I would like to have you attend this meeting as representative of your county, as there are matters of importance to come up which are of vital concern to you and to your county. Please take up this matter at once with your county judge and commissioners court with a view of having them pay the expenses of the trip, as your salary will certainly not justify the expense, and I do not like to insist upon you coming unless the commissioners court will pay the expenses. Moreover, the trip is in the interest of your county and will redound to a betterment of its sanitary conditions.

I am enclosing, herewith, copy of queries and you will be expected to hand in a written report covering these subjects. Do not enter into too much detail, but give me a short concise report.

I have perfected arrangements whereby all health officers can secure accommodations at Epworth Inn, right on the bay shore, at reasonable rates. In case you have not already notified me to reserve you a room, please do so at your earliest convenience, if you contemplate going.

Yours very truly,
W. M. BRUMBY, M. D.,
State Health Officer.

The following subjects are to be discussed at the Annual Conference of Texas Health Officers and Boards of Health, which meets in the auditorium, Epworth League ground, Corpus Christi, May 15th, 9 a. m.:

SANITATION.

What, if any, interest is being manifested by your people in sanitary affairs, such as improvement of jails, courthouses, slaughter houses, dairies, meat markets, and "clean up days" in cities and towns?

What towns or cities in your county have organized sanitary forces? Give number of men employed, including health officer and scavenger forces, and their duties. All city health officers to make separate report and to include appropriation for maintenance and no men.

PURE FOOD.

What effort, if any, outside larger cities is being made to have a milk and food inspection service?

QUARANTINE.

Report as far as possible number of cases, and any deaths that may have occurred, in your county, of the following diseases, for the year 1908; that is, since January 1st: Leprosy, smallpox, scarlet fever, diphtheria, epidemic cerebro-spinal fever, epidemic dysentery, typhoid, tuberculosis and trachoma.

Are the regulations on quarantine, isolation and disinfection complied with in every instance with smallpox, scarlet fever and diphtheria? Are the physicians of your town or city co-operating? Is the commissioners court (or city council, as the case may be) supporting you, and have they passed proper resolutions and ordinances authorizing you to immediately take control of these three diseases? Is compulsory vaccination one of the requirements for entrance to the public schools?

Are physicians reporting tuberculosis, and do you follow up these cases and disinfect the house in case of death or removal? Is there any perceptible increase in tuberculosis in your city or county, particularly as relates to the older residents, in the last five years?

(This paragraph is to be answered by those of you in the mosquito territory of tne State.) Are your people alive to the necessity of mosquito extermination, screening their houses and cisterns, and interested in better drainage? Do you favor not placing on a quarantine were you assured that there were only one or two imported cases of yellow fever in a given town or city in our State, and further assured that the disease was recognized early and all possible precautions taken from the beginning, such as screening and fumigation of house infected as well as houses in same block? Do you think that your constituents have sufficient confidence in you and your State Health Officer to listen to reason, and to stand for a "safe and a sane" method of protection, once assured of the above facts?

Have you read the proposed sanitary code of unincorporated towns, and do you think that the citizens in your unincorporated towns can be interested in incorporating for sanitary purposes under the law governing such?

VITAL STATISTICS.

What approximate per cent of births and deaths is being reported to your county clerk at present? What percentage of doctors are reporting births and deaths? Do midwives report births any better than physicians?

Now, doctor, you will be limited to three minutes each in reading the above report, so try to limit yourself to about 800 words and confine yourself strictly to a report of conditions and the subjects enumerated, and not go into detail. These reports are to be published later, so put same in writing, and in case your report can not be read in the allotted time you will please submit same without reading when the name of your county is called.

In case no county physician is present, any city physician in addition to his own can make report for his county—a verbal one if he prefers.

